# Aberrant Early in Life Stimulation of the Stress-Response System Affects Emotional Contagion and Oxytocin Regulation in Adult Male Mice

**DOI:** 10.3390/ijms22095039

**Published:** 2021-05-10

**Authors:** Giovanni Laviola, Ludovica Maria Busdraghi, Noemi Meschino, Carla Petrella, Marco Fiore

**Affiliations:** 1 Reference Centre for Behavioral Sciences and Mental Health, Istituto Superiore di Sanità, Viale Regina Elena, 299, 00161 Rome, Italy; busdraghi.1460902@studenti.uniroma1.it (L.M.B.); meschino.1650735@studenti.uniroma1.it (N.M.); 2 Institute of Biochemistry and Cell Biology, IBBC, CNR, Viale del Policlinico 155, 00161 Rome, Italy; carla.petrella@cnr.it (C.P.); marco.fiore@cnr.it (M.F.)

**Keywords:** early risk factors, stress, social disorders, empathy for pain, HPA, oxytocin

## Abstract

Results over the last decades have provided evidence suggesting that HPA axis dysfunction is a major risk factor predisposing to the development of psychopathological behaviour. This susceptibility can be programmed during developmental windows of marked neuroplasticity, allowing early-life adversity to convey vulnerability to mental illness later in life. Besides genetic predisposition, also environmental factors play a pivotal role in this process, through embodiment of the mother’s emotions, or via nutrients and hormones transferred through the placenta and the maternal milk. The aim of the current translational study was to mimic a severe stress condition by exposing female CD-1 mouse dams to abnormal levels of corticosterone (80 µg/mL) in the drinking water either during the last week of pregnancy (PreCORT) or the first one of lactation (PostCORT), compared to an Animal Facility Rearing (AFR) control group. When tested as adults, male mice from PostCORT offspring and somewhat less the PreCORT mice exhibited a markedly increased corticosterone response to acute restraint stress, compared to perinatal AFR controls. Aberrant persistence of adolescence-typical increased interest towards novel social stimuli and somewhat deficient emotional contagion also characterised profiles in both perinatal-CORT groups. Intranasal oxytocin (0 or 20.0 µg/kg) generally managed to reduce the stress response and restore a regular behavioural phenotype. Alterations in density of glucocorticoid and mineralocorticoid receptors, oxytocin and µ- and κ-opioid receptors were found. Changes differed as a function of brain areas and the specific age window of perinatal aberrant stimulation of the HPA axis. Present results provided experimental evidence in a translational mouse model that precocious adversity represents a risk factor predisposing to the development of psychopathological behaviour.

## 1. Introduction

Beside genetic background, an immature organism acquires developmental plasticity, basically through epigenetic modifications, since the very first phases of life, to rapidly adapt its phenotype in response to external environmental cues. The latter are relevant to the probable environment the mature organism will have to face, in order to optimize the chances of survival [1,2]. This adaptation may occur at the expense of a predisposition for stress-related diseases and psychiatric disorders (in humans), which may present clinical manifestations at different ages [3,4,5,6].

An extensive literature states that during the perinatal period, intense emotions and variations in the hormonal milieu of the mother can have a fundamental role in shaping the neurophysiologic systems of the offspring. Premature and marked activation of the stress axis, during early ontogenetic windows in the offspring (e.g., due to stressors and/or direct stimulation through aberrant levels of glucocorticoids) [7], have been shown to represent a predisposing factor towards vulnerability to the development of psychiatric symptoms such as conduct disorder and autism spectrum disorder [8,9,10,11].

Social motivation and actual social interactions result to be disrupted in adulthood, as a consequence of prenatal, neonatal and peripubertal exposure to physical stressors in both mice and rats [12,13]. Pregnancy and *postpartum* periods are characterised by fluctuations in the levels of pregnancy-related hormones, due to changes in their mechanisms of regulation [14]. Maternal hypothalamic-pituitary-adrenal (HPA) axis responds with different reactivity during pregnancy and lactation, and specific adaptations of both basal and stress-induced HPA activity occur during the reproductive cycle, in order to fulfil the demands of the offspring [15,16]. The most marked changes are the increase in basal secretion and the attenuation of the neuroendocrine response to stress. Interestingly, the hormones that are normally released in response to stress, and which show suppressed responses during lactation (oxytocin, prolactin, ACTH and corticosterone) are the same released in response to suckling. During the first ten postnatal days, a stress hyporesponsive period (SHRP) has been reported in rat and mouse pups. Generally, immature rodents are naturally kept less responsive to stress stimuli coming from the environment [17,18]. Indeed, a regular neurobehavioural development requires a general picture of low functional activation on both mother and offspring during the perinatal life. This finding may underlie an adaptive change that occurs as a protective response, since exposure to stress-even induced by synthetic glucocorticoids intake-can affect the development of physiological systems in the offspring, resulting in increased susceptibility to affective disorders in adulthood [17,18,19].

This evidence has been correlated to the transfer of maternal hormones to the foetus. In nonpathological situations placental enzyme 11 beta-hydroxysteroid dehydrogenase type 2 (11 beta-HSD-2) inactivates glucocorticoids [20], preventing the foetus to be exposed to high maternal glucorticoids. Under conditions of severe stress exposure, such protective mechanism cannot subserve its role leading the foetus to be exposed to abnormal levels of corticosterone [21]. Studies on induced maternal hypercorticosteronemy in rats, using a solution of corticosterone hemisuccinate administered through drinking water during late gestation or early lactation showed how glucocorticoids influence neural development and physiology in the offspring [19,20,21]. Prenatal glucocorticoid exposure in rats is associated later in life with impaired glucose tolerance, abnormalities of the hypothalamo–pituitary–adrenal axis and behaviour. In rats’ adult brain, elevated titres of circulating corticosterone appear capable of downregulating glucocorticoid receptor concentrations [22].

It has been shown that exposure to a synthetic glucocorticoid in late gestation, alters MR/GR ratio in the hippocampus [23]. This is an example of how external factors that have glucocorticoid or mineralocorticoid receptors as a potential target may induce changes in the response of the developing brain. In particular, this occurs among areas involved in the regulation of the HPA axis and behaviour, such as amygdala, hippocampus and hypothalamus [21,23,24,25]. Postnatal corticosterone exposure, moreover, seems to have a dose-dependent effect on the progeny. Indeed, it has been shown that low corticosterone supplementation may favour resilience in the offspring in terms of activation of the hypothalamus-pituitary-adrenal (HPA) axis. Whereas, high doses have a maladaptive outcome, leading to an augmented stress reactivity [26,27,28].

Environmental factors that affect the development of neuroendocrine structures are linked to perturbed neurocognitive functions and psychiatric conditions such as conduct disorder, which are characterised by profound alterations of empathic ability to recognize distress in other together with abnormal socialisation practices [29], as well as augmented susceptibility to the occurrence of aggressive episodes, and learning deficits [30]. Individuals affected by major psychiatric symptoms, as reported in the Diagnostic Statistical Manual (DSM-V) [31], show profound alterations of social domain, including a reduction of affective and cognitive aspects of empathy behaviour. A coherent characterisation of empathy among many animal species can be based on the Perception-Action model elaborated by Preston and de Waal [32]. Perception-action mechanisms are particularly important if mother-offspring bond is considered. This theoretical framework manages fine in combining behavioural data on empathy with data from physiology and functional neuroanatomy. Brothers suggested that experiencing an observed emotion is directly related with the ability to understand that state [33].

The empathic response (emotional contagion) has been shown in both humans and animal models to be inhibited under stress conditions by the groups of Sapolsky and Mogil [34], while it is significantly released upon pharmacological inhibition of glucocorticoids synthesis. In this framework, we specifically investigated the adult offspring of mouse dams exposed to increased concentrations of corticosterone in drinking water, during two critical age windows of offspring development-namely, late gestation and early lactation. This was aimed to reach an abnormal stimulation of the HPA axis of the mother, and thus of the still immature offspring.

Social dysfunction represents a major symptom in schizophrenia, autism spectrum disorders, conduct disorder and other psychiatric conditions. An increasing amount of evidence suggests that alterations of the oxytocinergic system may be involved in the onset of these psychiatric diseases [35,36,37]. This system begins to develop in utero, and it has been demonstrated that perinatal stress can cause alteration both of the Oxytocin (OXT) and the Vasopressin (VP) signalling pathways [38]. These alterations have been correlated with long-term effects such as social behaviour impairments, as well as increased aggression in later life. However, the precise role of early life adversities and stress in the development of the oxytocinergic system has still to be clarified [39].

The neuropeptide oxytocin has received great research and clinical interest in line with its demonstrated key role in social cognition, and social behaviours. Oxytocin seems to lead to an increase in pro-social and empathic behaviours, and a reduction in anxious, aggressive and compulsive behaviours [40,41,42,43]. Several preclinical and clinical studies are thus underway to investigate the effectiveness of exogenous oxytocin administrations in improving the symptoms associated with such pathological conditions [44,45]. Effects of this neurotransmitter, thus, depend on the levels of expression of oxytocin receptors in the brain that turn out to be dependent on genetic and epigenetic factors that act at the level of the gene coding for the receptor itself [36,46,47].

With the aim of developing an experimental preclinical model, we studied the male offspring during adulthood for changes in the HPA axis function and the response to acute restraint stress, comorbid for profiles of social competence, and emotional contagion [43,48]. This was in consideration of the demonstrated strict interplay between activation of HPA axis function and empathy capacity in humans and rodent models [34]. Namely, levels of emotional contagion for pain in other, were measured. Notably, fine-grained behavioural scoring was carried out to identify normal and abnormal profiles. We relied on preclinical studies reporting the presence of behaviours referable to empathy in rodents [49]. Mice were evaluated by means of a battery of behavioural tests focused on profiles of social motivation and social novelty preference. We complemented the study with the assessment of the modulatory in vivo effects of intranasal administration of oxytocin [43]. Considering the sex-biased occurrence of psychiatric conditions such as conduct disorder and autism spectrum disorders [9,10,11] the male offspring was investigated.

Another aspect that has been taken into consideration was a molecular investigation of the gene expression for glucocorticoid, mineralocorticoid, μ-opioid, κ-opioid and oxytocin receptors in behaviourally relevant brain areas of interest. In order to investigate the molecular correlations scored for the different groups, prefrontal cortex, hypothalamus and hippocampus were chosen as areas of interest, since they are involved in the regulation of the behavioural output with respect to the considered parameters. Glucocorticoid receptors (GR) and mineralocorticoid receptors (MR) density and their ratio were evaluated to confirm the specific long-term consequences of perinatal experimental manipulation on main components of the HPA axis. Oxytocin receptors (OXTR) characterisation has been proposed in order to account for the differential reactivity to this drug that is likely to be observed among the different perinatal groups. In addition, μ-opioid receptors (MOR) and κ-opioid receptors (KOR) density was considered as well, given the opposite role exerted by these receptors in the modulation of social behaviour [50,51].

## 2. Results

**Figure 1:**
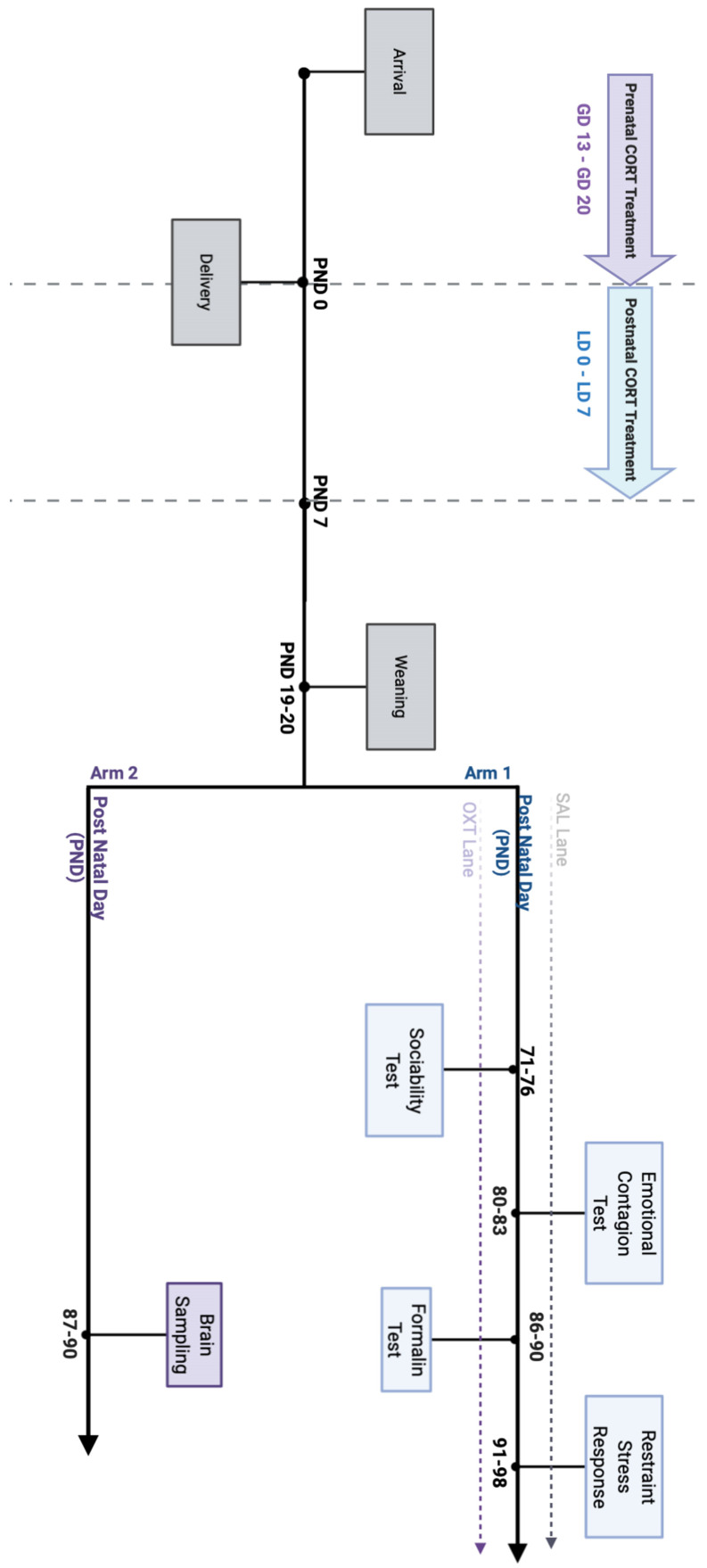
Timeline. Schematic representation of the experimental timeline for all the subjects included in the test battery.

Subjects from the present study were split into two experimental arms (see the Section 4 Materials and Methods section for additional information). All behavioural data refer to animals pertaining to arm 1, while molecular analyses were performed on subjects assigned to arm 2 (Figure 1).

### 2.1. Body Weight Monitoring

Mice body weight was monitored during the whole test battery. A significant main effect of perinatal treatment was found (F_2,42_ = 7.450, *p* = 0.002), with body weight as a whole slightly reduced in both PreCORT and PostCORT subjects, compared with AFR controls. Oxytocin administration before testing (F_1,42_ = 1.447, *p* = 0.236) and interaction between treatment and drug (F_2,42_ = 1.215, *p* = 0.307) had no relevant effects.

### 2.2. Plasma Corticosterone Assessment

To confirm and extend the consequences of the abnormal stimulation of the HPA axis reached by the exposure of immature mice to corticosterone (the major effector of stressors) during two critical early phases of development-namely, late gestation or early postnatal life-HPA axis reactivity of adult mouse offspring to acute restraint stress was evaluated. Blood samples were collected at different time-points from each subject, and repeated measures ANOVA revealed-as expected-a significant effect of time (F_3,126_ = 46.004, *p* < 0.001). Inspection of the resultant curves shows how at t0, both PreCORT and PostCORT subjects (saline administered) were characterised by already increased basal levels of plasma corticosterone, compared with the AFR control group. Corticosterone levels increased markedly during the restraint procedure, due to the activation of the physiological stress response. The peak was reached at t60, and this appeared quite higher in both PreCORT and PostCORT saline-administered subjects compared with the corresponding AFR group; at t120 lower levels of corticosterone were observed, as they already began to return to basal levels. When considering the last interval in the hormonal curve, it was evident a marked delay in turning back to baseline levels after the peak, in both PreCORT and PostCORT subjects, compared with the control AFR group. Thus, the feedback process appeared altered in subjects exposed to corticosterone during the perinatal period.

**Figure 2:**
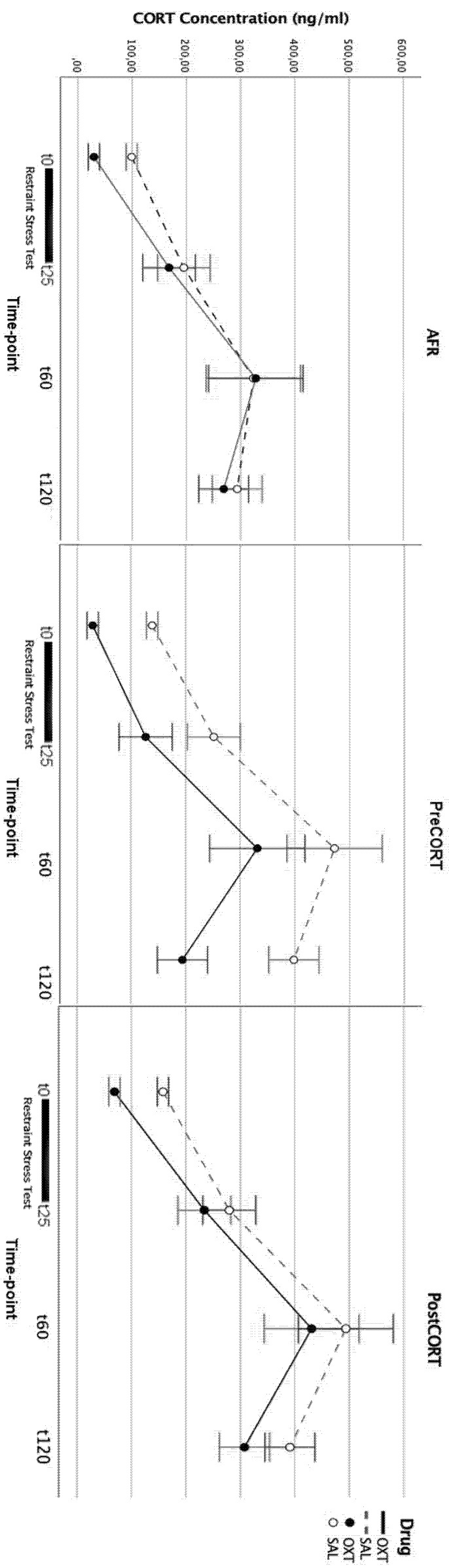
Plasma corticosterone concentration. Plasma corticosterone concentrations Subjects: AFR-SAL, AFR-OXT, PreCORT-SAL, PreCORT-OXT, PostCORT-SAL and PostCORT OXT (*n* = 8). Blood samples were collected before a 25-min restraint stress (t0), immediately after (t25) 35 and 95 min later (t60 and t120). Subjects were administered either intranasal saline or oxytocin (20 μg/mL) 10 min before the test in a volume of 2 μL. Error bars indicate mean ± SEM.

Mice were also administered either intranasal oxytocin or saline before testing. Oxytocin as expected, had a positive modulatory effect on the HPA axis, by generally reducing its activation. At t60 (peak) PreCORT and PostCORT oxytocin-administered mice had lower levels of corticosterone, compared with the saline-treated counterpart. It is possible to notice that oxytocin-administered PreCORT subjects had a slightly faster decline of corticosterone concentration than AFR and PostCORT subjects upon the same treatment (Figure 2).

**Figure 3:**
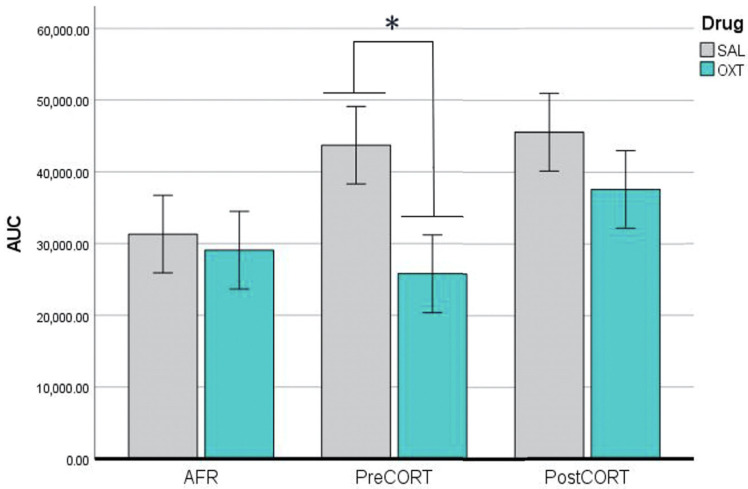
Area Under the Curve (AUC). The AUC was calculated following the trapezoidal rule for plasma corticosterone concentration in subjects that were involved in the Restraint Stress test. Subjects: AFR-SAL, AFR-OXT, PreCORT-SAL, PreCORT-OXT, PostCORT-SAL and PostCORT-OXT (*n* = 8 for each group) (* *p* < 0.05). Error bars indicate mean ± SEM.

In addition, the Area Under the Curve (AUC) was evaluated for each subject following the trapezoidal rule. ANOVA performed for AUC indicated that perinatal treatment, per se, was not significant (F_2,42_ = 2.255, *p* = 0.117). However, significant effects of drug administration were observed (F_1,42_ = 4.506, *p* = 0.040). The post hoc analysis carried out within a not significant interaction between perinatal treatment and drug (F_2,42_ = 1.080, *p* = 0.349) indicated that oxytocin administration consistently reduced the stress response in the PreCORT group (Figure 3). A slight drug modulation appeared in the other two perinatal groups.

### 2.3. Sociability Test

This test was performed to investigate how the perinatal treatment affected different aspects of sociability in adult mice, specifically in terms of social motivation (Episode 2, choice between the social stimulus vs. the empty one) and of social novelty preference (Episode 3, the choice between a novel unfamiliar conspecific and a familiar one, previously encountered during Episode 2).

#### 2.3.1. Episode 1—Habituation Phase

During Episode 1, mice general locomotion within the empty three-chambered apparatus, was evaluated in order to monitor for unspecific consequences due to perinatal treatment or effects driven by oxytocin administration before testing. Two-way ANOVA revealed no differences due to perinatal treatment (F_2,42_ = 1.093, *p* = 0.344) or drug administration (F_1,42_ = 0.861, *p* = 0.359); nor their interaction as well (F_2,42_ = 0.004, *p* = 0.996) (Data not shown).

#### 2.3.2. Episode 2—Social Preference

**Figure 4:**
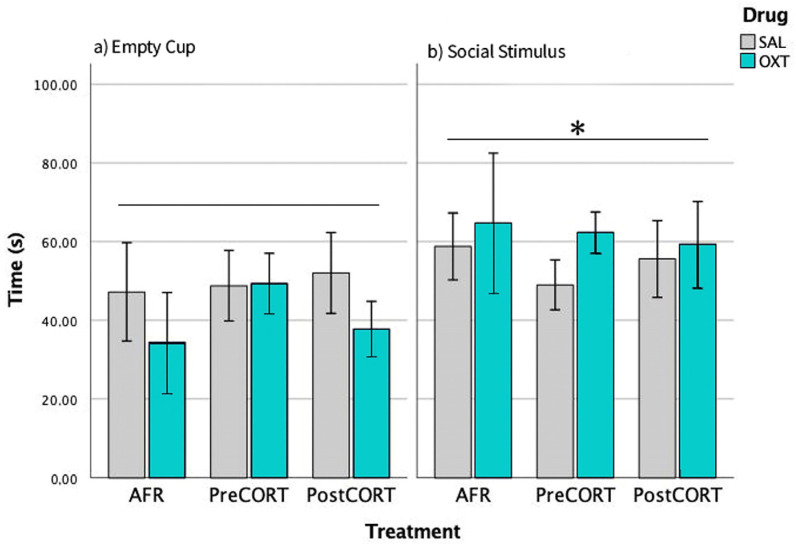
Sociability test, Episode 2. Comparison between time spent near the empty cup vs. cup containing the social stimulus. Subjects: AFR-SAL, AFR-OXT, PreCORT-SAL, PreCORT-OXT, PostCORT-SAL and PostCORT-OXT (*n* = 8 for each group). During Episode 2, a general effect of the position was observed: all mice, regardless of the perinatal treatment and the drug administered before testing, spent increased time interacting with the social stimulus (* *p* < 0.05). Error bars indicate mean ± SEM.

Preference for social stimulus was scored. ANOVA revealed a main effect of position (F_1,42_ = 5.671, *p* = 0.022): globally, if the perinatal treatment and drug administration are not taken into account, we found subjects spent more time close to the cup containing the social stimulus over the empty one. This result confirmed the validity of the test paradigm (Figure 4). Perinatal treatment did not apparently affect this parameter (F_2,42_ = 0.008, *p* = 0.931), per se. The same was for drug administration (F_1,42_ = 0.015, *p* = 0.985) and interaction between the two factors (F_2,42_ = 0.370, *p* = 0.693).

#### 2.3.3. Episode 3—Social Novelty Preference

**Figure 5:**
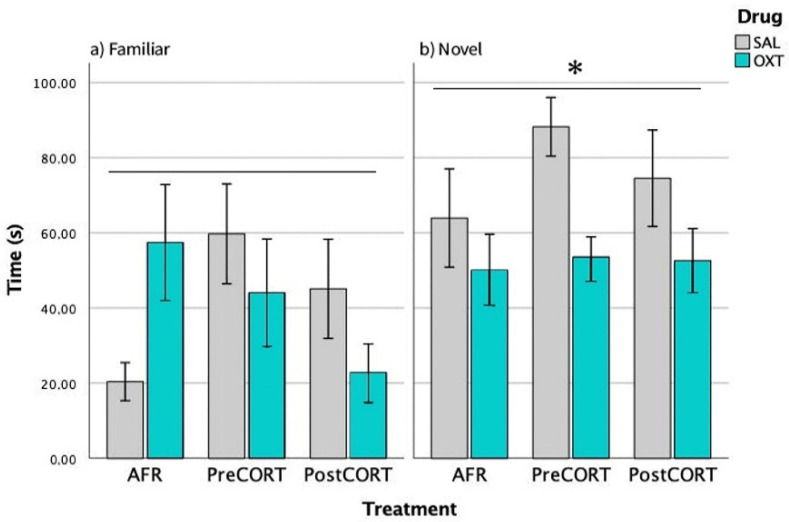
Sociability test, Episode 3. Comparison between time spent near the familiar vs. novel social stimulus. Subjects: AFR-SAL, AFR-OXT, PreCORT-SAL, PreCORT-OXT, PostCORT-SAL and PostCORT-OXT (*n* = 8 for each group). During Episode 3, all mice exhibited a general preference for the novel social stimulus over the familiar one (* *p* < 0.05). Error bars indicate mean ± SEM.

Preference by experimental mice for the novel social stimulus over an already familiar one was scored. As expected, all preferred interacting with the unfamiliar social stimulus compared with the familiar one-main effect of position (F_1,42_ = 10.920, *p* = 0.002). This finding confirms again the validity of the test paradigm (Figure 5). Perinatal treatment reached significance on this profile (F_2,42_ = 3.904, *p* = 0.055); while no effects due to drug administration per se were observed (F_1,42_ = 2.089, *p* = 0.136). The interaction between perinatal treatment and drug was statistically significant (F_2,42_ = 3.806, *p* = 0.030).

Separate ANOVA focused on data related to the Familiar social stimulus only indicated that perinatal treatment, per se (F_2,42_ = 1.943, *p* = 0.155), and drug (F_1,42_ = 0.218, *p* = 0.643) were not significant, while their interactions just missed significance (F_2,42_ = 2.605, *p* = 0.085). A close inspection of Figure 5, (left panel) and the post hoc suggested increased social levels in both saline-administered Pre- and PostCORT subjects compared to corresponding AFR controls. Oxytocin administration significantly increased, as expected, the response in the AFR group, while it seemed to contrast the profile in both perinatal CORT groups. With respect to time spent with the Novel social stimulus only (right panel), separate ANOVA indicated a main effect due to drug administration (F_1,42_ = 3.904, *p* = 0.054) and a significant interaction between perinatal treatment and drug (F_2,42_ = 3.806, *p* = 0.030). An abnormally elevated preference for the novel social stimulus characterised saline-administered PreCORT subjects, compared with AFR controls. A similar tendency was also apparent for the PostCORT group. Intranasal oxytocin, as a whole, reduced the profile, and this effect occurred more consistently in the PreCORT group.

### 2.4. Emotional Contagion Assay

#### 2.4.1. Observers’ Paw-Licking Behaviour

For the analysis of time spent performing paw-licking behaviour by Observer mice, we also considered the specific body posture adopted (see Materials and Methods). ANOVA considering body posture x perinatal treatment x drug did not evidence major changes due to perinatal treatment., while oxytocin induced a significant increase of paw-licking in the PreCORT group only, with the finding of a significant perinatal treatment x drug interaction (F_2,42_ = 3.749, *p* = 0.032) in the ANOVA. As a whole a sort of preference emerged, with mice from most groups showing a tendency towards the adoption of the in-back posture, compared to the in-front one (F_1,42_ = 3.635, *p* = 0.063), This profile appeared particularly released upon oxytocin administration, as confirmed by the finding of a significant interaction of drug and body posture (F_1,42_ = 6.591, *p* = 0.014). (For the sake of space, data are not shown).

**Figure 6:**
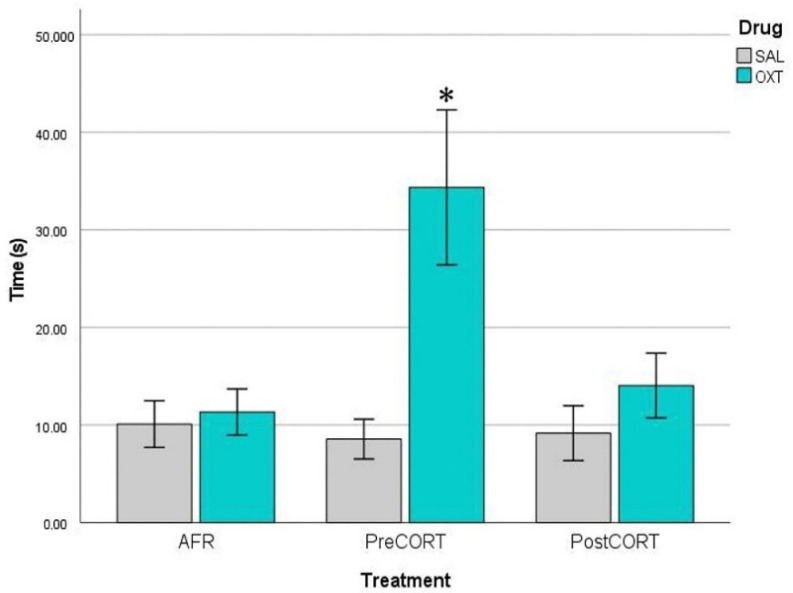
Observers’ Paw Licking behaviour. Time spent in paw-licking behaviour by Observer mice (*n* = 8 for each group), regardless the posture adopted towards the Demonstrator mouse. Oxytocin administration had a significant effect on PreCORT subjects (* *p* < 0.05), increasing the rate of licking behaviour. Error bars indicate mean ± SEM.

For time spent in paw-licking and presenting the in-back posture to the Demonstrator (Figure 6), ANOVA revealed no effects of perinatal treatment per se (F_2,42_ = 1.369, *p* = 0.265), while a main effect of drug administration was observed (F_1,42_ = 9.024, *p* = 0.045). Close inspection of Figure 6 and the post-hoc suggested a tendency towards deficient rate of paw-licking by both PreCORT and PostCORT saline administered mice in comparison with corresponding AFR controls. Further, the interaction of drug and perinatal treatment (F_2,42_ = 3.352, *p* = 0.045) was significant. Notably, oxytocin administration increased the rate of paw-licking, which is in line with this measure as an index of emotional contagion, and reverted the deficient profile, with PreCORT mice again being the most responsive group.

#### 2.4.2. Observers’ Self-Grooming

**Figure 7:**
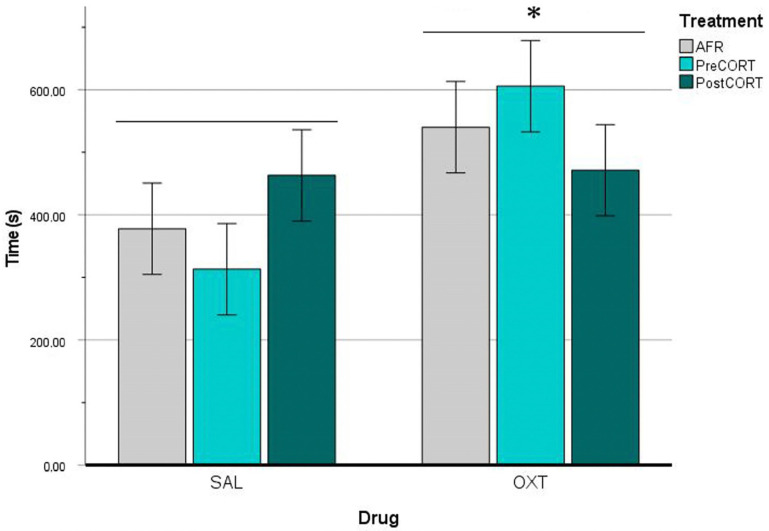
Self-grooming behaviour of Observer mice. Time spent by Observers (*n* = 8 for each group) in self-grooming activity during the whole test. Regardless perinatal treatment, oxytocin administration globally, resulted in increased rates of self-grooming (* *p* < 0.05) during the emotional contagion assay. Error bars indicate mean ± SEM.

ANOVA carried out on data of self-grooming behaviour exhibited by the Observer mice, revealed no main effects of perinatal treatment, per se (F_2,42_ = 0.008, *p* = 0.992). However, drug administration resulted in a significant main effect (F_1,42_ = 6.711, *p* = 0.013), which was independent from the interaction between perinatal treatment and drug (F_2,42_ = 1.900, *p* = 0.162). Globally, Observer mice that received oxytocin before the test exhibited increased rates of self-grooming (Figure 7) during the session of the emotional contagion assay.

#### 2.4.3. Observers’ Hole-Probe Behaviour

**Figure 8:**
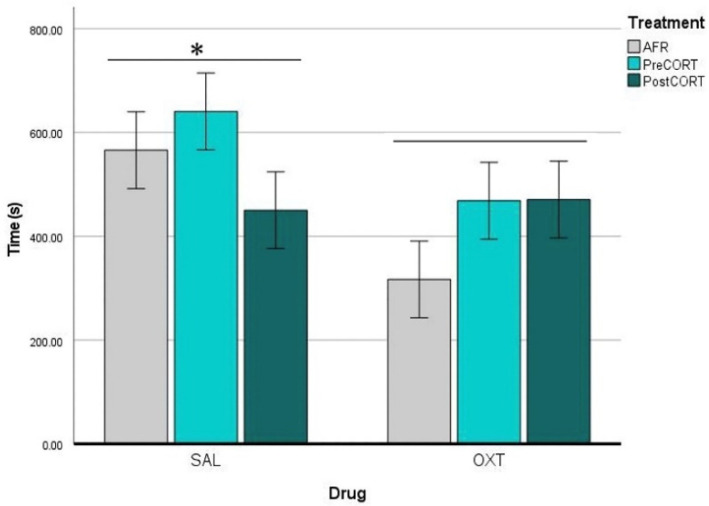
Hole-probe behaviour by Observer mice. Levels of hole-probe behaviour (nose pokes through the holes on the separator) exhibited by Observers (*n* = 8 for each group) towards the Demonstrator mouse were consistently reduced in mice that received intranasal administration of oxytocin before the test (* *p* < 0.05). This effect was independent from the interaction between perinatal treatment and drug. Error bars indicate mean ± SEM.

During the emotional contagion assay, Observer mice had the opportunity to interact with the Demonstrator mouse through small holes on the clear separator subdividing the two sides of the apparatus. Through these holes, an easy exchange of vocalizations and body odours could take place. Time spent in Hole-probe behaviour consisted of several attempts of physical interaction, which were exhibited repeatedly during the session by Observer mice towards the Demonstrator companion. Inspection of the videos and repeated measures ANOVA showed that this behaviour had a high and constant rate and was not subjected to habituation. Actually, this could not be satisfied due to the presence of the separator. In this view, it is not surprising that oxytocin administration contrasted this apparently compulsive activity, in most of the subjects. Indeed, regardless any effects of perinatal treatment (F_2,42_ = 1.341, *p* = 0.272), ANOVA revealed a main effect of drug: oxytocin exerted a negative modulation (Figure 8), by substantially decreasing the rate of Hole-probe behaviour by Observer mice (F_1,42_ = 4.891, *p* = 0.032). Interaction between perinatal treatment and drug (F_2,42_ = 1.760, *p* = 0.184) was not significant.

#### 2.4.4. Demonstrators’ Paw-Licking Behaviour

During the course of the emotional contagion assay, the Demonstrator mouse had the possibility to interact freely with each of the three Observer mice from behind the clear separator, subdividing the two sides of the testing apparatus. We considered whether or not the performance of Demostrator mice was indirectly affected by the individual characteristics of individual Observer companions involved in social interaction. The duration of time spent in each behaviour by the Demonstrator mouse, in proximity to each of the three Observer companions was expressed as a percentage of its total activity.

**Figure 9:**
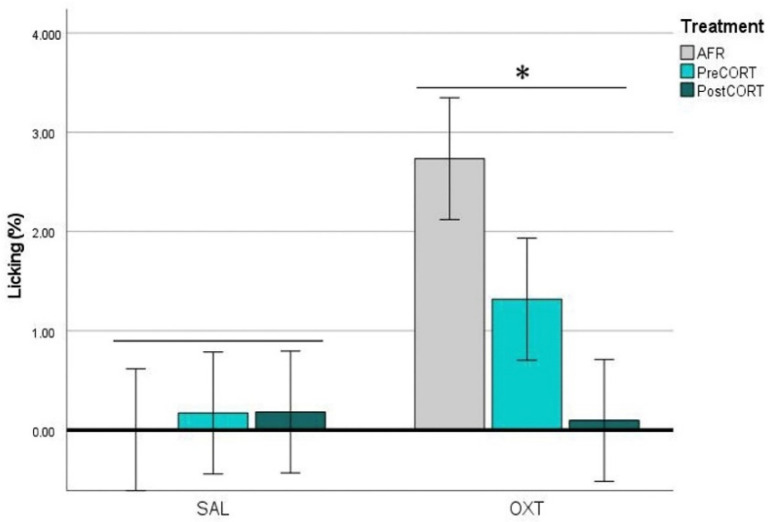
Percentage of paw-licking exhibited by the Demonstrators. Observer mice (*n* = 16) were allowed to interact in proximity to the three Observer companions, each belonging to a different perinatal treatment. Half of the Observers received intranasal administration of oxytocin; the other half received saline. As a whole, Demonstrators showed increased levels of paw-licking when close to oxytocin-administered Observer companions (* *p* < 0.05). Error bars indicate mean ± SEM.

In response to the injection of a high formalin dose (1%), a prompt and marked profile of paw-licking behaviour was released. No effects of perinatal treatment were found (F_2,42_ = 2.003, *p* = 0.148). Interestingly, however, if the drug received by the Observer companions before testing was oxytocin, this seemed to affect consistently levels of emotional contagion exhibited by the Demonstrator. Indeed, compared to the licking rate exhibited when in proximity to saline-administered companions, the Demonstrators appeared specifically more involved in paw-licking when found in proximity to the Observer companions that were administered oxytocin. This was supported by the finding of a main effect of drug (F_1,42_ = 6.355, *p* = 0.016), and a just missing significant interaction of perinatal treatment and drug (F_2,42_ = 2.639, *p* = 0.083), in the ANOVA. Indeed, the indirect influence of oxytocin administration appeared specifically attributable to changes in the AFR group (Figure 9).

#### 2.4.5. Demonstrators’ Self-Grooming

**Figure 10:**
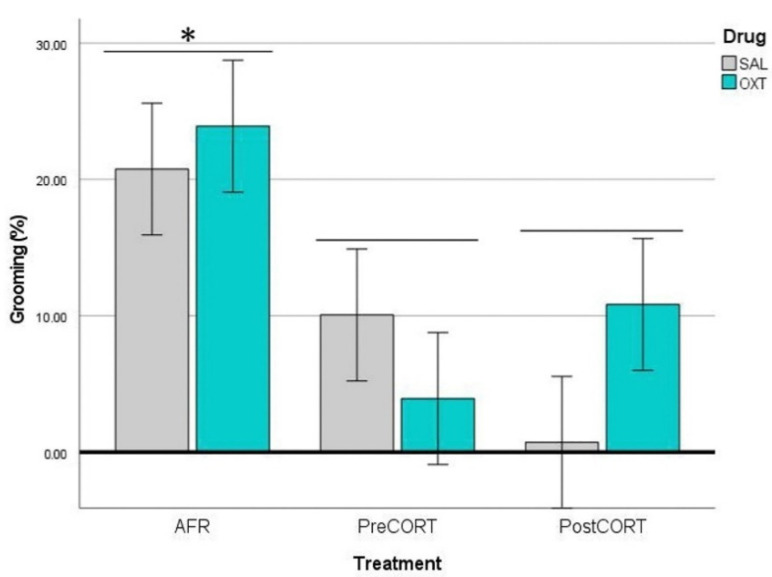
Percentage of self-grooming exhibited by Demonstrator mice. Demonstrators (*n* = 16) when found in proximity of three Observer companions, each belonging to a different perinatal treatment. The Observers were administered either intranasal oxytocin or received saline. Demonstrator mice exhibited, as a whole, an important reduction in the rate of self-grooming when close to both PreCORT and PostCORT Observers than to the AFR control group (* *p* < 0.05). This was regardless the drug Observer companions received before the test. Error bars indicate mean ± SEM.

ANOVA carried out on data of self-grooming behaviour exhibited by the Demonstrator mice, revealed a clear even if only indirect influence of the specific perinatal treatment to which Observer companions belonged to (F_2,42_ = 8.159, *p* = 0.010). As a whole, consistently reduced rates of self-grooming were exhibited by Demonstrator mice when in proximity to Observer companions from both PreCORT and PostCORT groups than to the AFR control group (Figure 10). This profile was apparently independent from drug effects (F_1,42_ = 0.205, *p* = 0.653) or the interaction between perinatal treatment and drug (F_2,42_ = 1.641, *p* = 0.206).

#### 2.4.6. Demonstrators’ Hole-Probe Behaviour

**Figure 11:**
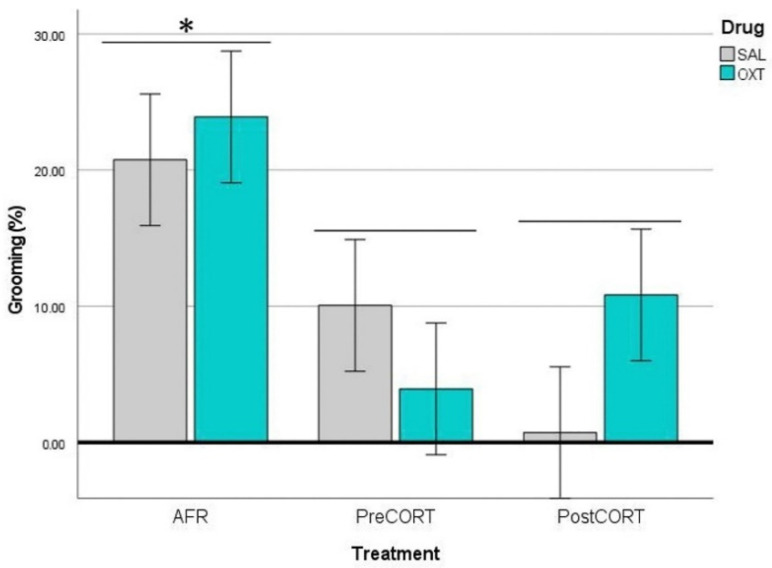
Percentage of Hole-probe behaviour exhibited by Demonstrators. Demonstrator mice (*n* = 16) were free to spend time close to each of the three Observer companions, each one belonging to a different perinatal treatment. Observers received either intranasal administration of oxytocin before testing or received saline. Demonstrator mice spent a significant higher amount of time interacting with PostCORT subjects, in comparison with AFR subjects (* *p* < 0.05). Intranasal oxytocin administration to the Observers did not apparently affect this profile. Error bars indicate mean ± SEM.

ANOVA revealed again significance for an indirect influence of the perinatal treatment, which represents an individual characteristic of the three Observer mice (F_2,42_ = 3.739, *p* = 0.032). Specifically, Demonstrator mice spent as a whole-increased amount of time interacting with Observer PostCORT companions than with the AFR controls (* *p* < 0.05). Drug administration (F_1,42_ = 0.724, *p* = 0.340) and the interaction between treatment and drug (F_2,42_ = 2.177, *p* = 0.126) led to not significant effects (Figure 11).

### 2.5. Profiles of Pain Sensitivity and of Response to Oxytocin in a Social vs. a Non-Social Context

**Figure 12:**
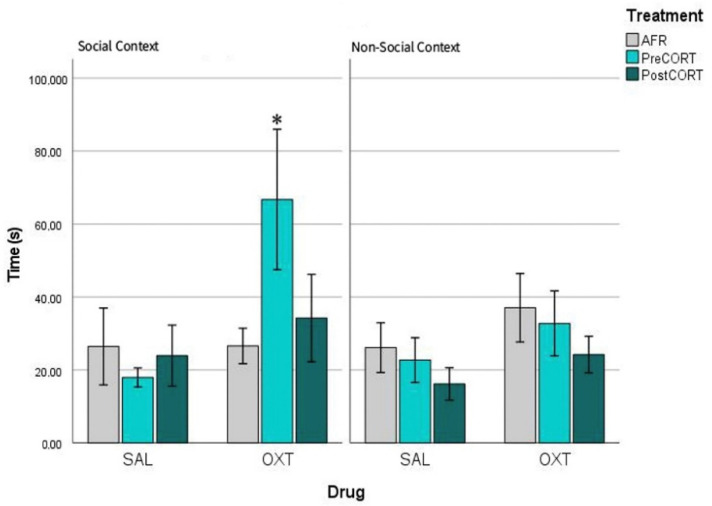
Comparison of licking rate exhibited by mice in a social vs. a non-social context. Mice that previously acted as Observers (*n* = 8 for each group) in the Emotional Contagion assay (social context) and, to the aims of the formalin test (non-social context), received an injection of formalin (low dose, 0.2%) in a hind paw. Inspection of the results showed how oxytocin administration had only marginal or negligible effects in a non-social context. In contrast, a specific increased sensitivity to drug effects emerged in the social context. Again, this increased drug sensitivity was specific for the PreCORT subjects (* *p* < 0.05). Error bars indicate mean ± SEM.

Comparison of paw-licking rate exhibited by mice that served previously as Observers in the social context (emotional contagion assay) vs. a non-social context (formalin test). This within-subject analysis also allowed to investigate whether mice of three perinatal groups exhibited differences when tested for sensitivity to pain and response to oxytocin, in a social context vs. a non-social context (Figure 12). The interaction between perinatal treatment, drug and test condition just missed significance (F_2,42_ = 2.808, *p* = 0.070). As a whole, a slightly increased sensitivity to pain appeared associated to a social context, namely the emotional contagion assay (left panel), compared to the non-social context of the formalin test (right panel). Notably, this profile emerged specifically potentiated under the action of oxytocin effect in the social context. Regardless effects of perinatal treatment, per se (F_2,42_ = 1.357, *p* = 0.268), oxytocin administration slightly affected the response in all groups (F_1,42_ = 5.346, *p* = 0.026). Indeed, interaction between perinatal treatment and drug was not significant (F_2,42_ = 0.022, *p* = 0.978). Again, in line with the aims of the present study, this unique increased sensitivity to drug effects was unveiled thanks to the characteristic response of PreCORT group; notably when the latter was faced with a social context.

### 2.6. Molecular Analyses

Molecular analyses were performed on brains collected from AFR, PreCORT and PostCORT mice that were left undisturbed until sacrifice in adulthood (see Materials and Methods. In agreement with the relevant literature, analyses were performed on three different brain areas of interest: prefrontal cortex, hypothalamus and hippocampus.

#### 2.6.1. Glucocorticoid Receptors (GR)

**Figure 13:**
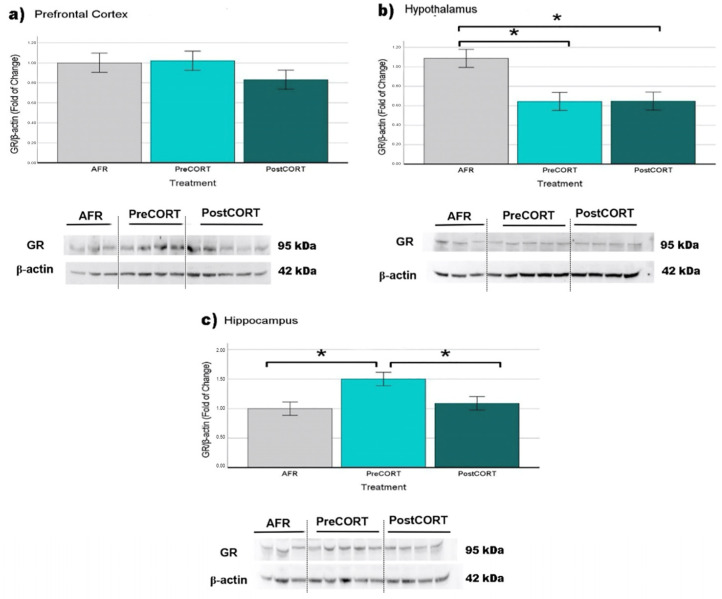
Glucocorticoid Receptors (GR) expression. Histograms and representative bands of GR expression in (**a**) prefrontal cortex, (**b**) hypothalamus and (**c**) hippocampus in AFR (*n* = 8, prefrontal cortex; *n* = 6, hypothalamus; *n* = 8, hippocampus); PreCORT (*n* = 8, prefrontal cortex; *n* = 6, hypothalamus; *n* = 8, hippocampus) and PostCORT (*n* = 8 prefrontal cortex; *n* = 6 hypothalamus; *n* = 8 hippocampus) subjects. Each lane corresponds to different mice. Data are presented as mean ± SEM (* *p* < 0.05).

Prefrontal Cortex: no significant differences (F_2,23_ = 1.174, *p* = 0.329) among perinatal treatment groups were observed (Figure 13a).

Hypothalamus: glucocorticoid receptors resulted to be significantly reduced as a consequence of perinatal treatment (F_2,17_ = 7.555, *p* = 0.005): PreCORT and PostCORT presented lower levels of receptors than AFR group (Figure 13b).

Hippocampus: perinatal treatment was significant (F_2,23_ = 5.465, *p* = 0.012) with the PreCORT group showing a remarkable increase in GR levels in comparison with other two experimental groups (Figure 13c). No differences were observed between PostCORT and AFR controls.

#### 2.6.2. Mineralocorticoid Receptors (MR)

**Figure 14:**
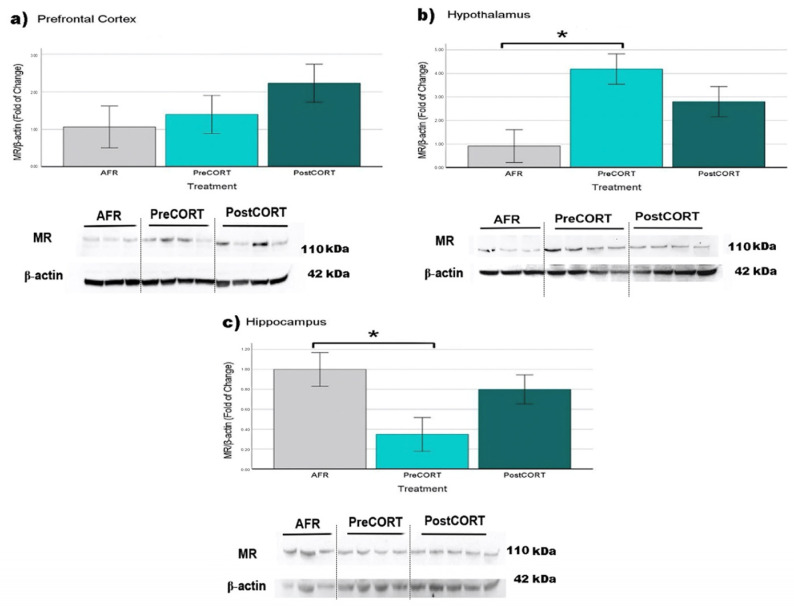
Mineralocorticoid receptors (MR) expression. Histograms and representative bands of MR expression in (**a**) prefrontal cortex, (**b**) hypothalamus and (**c**) hippocampus in AFR (*n* = 5, prefrontal cortex; *n* = 6, hypothalamus; *n* = 6, hippocampus); PreCORT (*n* = 6, prefrontal cortex; *n* = 7, hypothalamus; *n* = 6, hippocampus) and PostCORT (*n* = 6, prefrontal cortex; *n* = 7, hypothalamus; *n* = 8, hippocampus) subjects (* *p* < 0.05). Each lane corresponds to different mice. Data are presented as mean ± SEM (* *p* < 0.05).

Prefrontal Cortex: Density of MR was not affected by perinatal treatment (F_2,16_ = 1.313, *p* = 0.300), however, a tendency for PostCORT subjects to exhibit augmented levels, compared with AFR controls and the PreCORT group, can be noticed (Figure 14a).

Hypothalamus: A main effect of perinatal treatment (F_2,19_ = 5.933, *p* = 0.011) appeared, with PreCORT subjects being characterised by significantly reduced density of MR receptors, compared with AFR controls (Figure 14b).

Hippocampus: A main effect of perinatal treatment appeared (F_2,19_ = 3.930, *p* = 0.040). Mineralocorticoid receptors in this area were significantly lower in PreCORT subjects (Figure 14c), compared with the AFR group.

#### 2.6.3. µ-Opioid Receptors (MOR)

**Figure 15:**
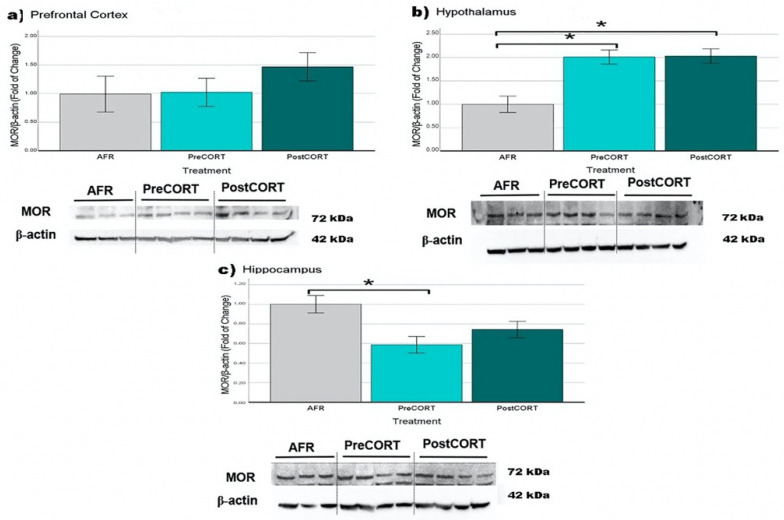
µ-Opioid Receptors (MOR) expression. Histograms and representative bands of MOR expression in (**a**) prefrontal cortex, (**b**) hypothalamus and (**c**) hippocampus in AFR (*n* = 5, prefrontal cortex; *n* = 6, hypothalamus; *n* = 7, hippocampus); PreCORT (*n* = 8, prefrontal cortex; *n* = 8, hypothalamus; *n* = 8, hippocampus) and PostCORT (*n* = 8, prefrontal cortex; *n* = 8, hypothalamus; *n* = 8, hippocampus) subjects (* *p* < 0.05). Each lane corresponds to different mice. Data are presented as mean ± SEM (* *p* < 0.05).

Prefrontal Cortex: PostCORT subjects exhibited a tendency towards increased levels of µ-opioid receptors compared with AFR and PreCORT groups (Figure 15a); however perinatal treatment failed the significance test (F_2,20_ = 1.055, *p* = 0.369).

Hypothalamus: A main effect of perinatal treatment (F_2,21_ = 12.487, *p* = 0.001) was found. µ-opioid receptors resulted significantly increased in both PreCORT and PostCORT subjects (Figure 15b), compared with the AFR control group.

Hippocampus: Relevant changes due to the perinatal treatment were observed in this area as well (F_2,22_ = 5.758, *p* = 0.011). PreCORT subjects showed a consistent reduction in levels of these receptors, compared with AFR mice (Figure 15c).

#### 2.6.4. κ-Opioid Receptors (KOR)

**Figure 16:**
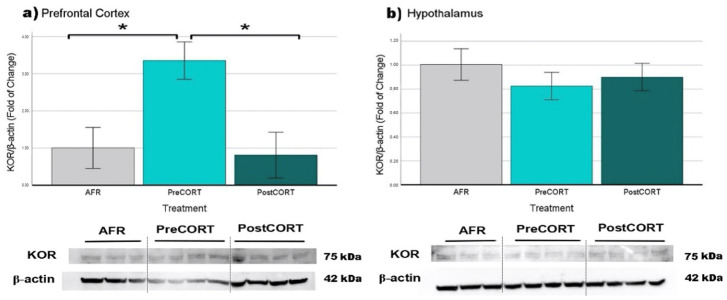
κ-Opioid Receptors (KOR) expression. Histograms and representative bands of KOR expression in (**a**) prefrontal cortex and (**b**) hypothalamus in AFR (*n* = 5, prefrontal cortex; *n* = 6, hypothalamus); PreCORT (*n* = 6, prefrontal cortex; *n* = 8, hypothalamus) and PostCORT subjects (*n* = 4, prefrontal cortex; *n* = 8, hypothalamus). Each lane corresponds to different mice. Data are presented as mean ± SEM (* *p* < 0.05).

Prefrontal Cortex: Relevant changes due to the perinatal treatment (F_2,14_ = 7.038, *p* = 0.010) were observed, with the PreCORT group showing significantly higher levels of KOR compared with AFR and PostCORT ones (Figure 16a). There were no differences between PostCORT and AFR groups.

Hypothalamus: No significant differences were found (F_2,21_ = 0.531, *p* = 0.596) among all three perinatal groups (Figure 16b).

#### 2.6.5. Oxytocin Receptors (OXTR)

**Figure 17:**
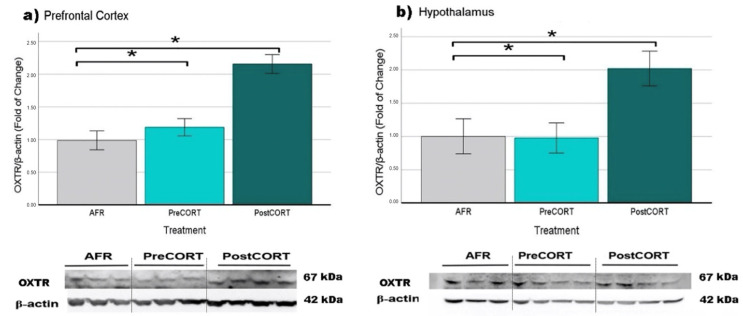
Oxytocin Receptors (OXTR) expression. Histograms and representative bands of OXTR expression in (**a**) prefrontal cortex and (**b**) hypothalamus in AFR (*n* = 5, prefrontal cortex; *n* = 6, hypothalamus); PreCORT (*n* = 6, prefrontal cortex; *n* = 8, hypothalamus) and PostCORT subjects (*n* = 5, prefrontal cortex; *n* = 6, hypothalamus). Each lane corresponds to different mice. Data are presented as mean ± SEM (* *p* < 0.05).

Prefrontal Cortex: A main effect of perinatal treatment was found (F_2,15_ = 18.843, *p* = 0.001), with significantly increased levels evidenced in the PostCORT group compared with AFR and PostCORT subjects (Figure 17a).

Hypothalamus: Similar results (F_2,19_ = 5.424, *p* = 0.015) were obtained in the hypothalamus: receptors levels in PostCORT subjects were significantly higher compared to AFR and PreCORT ones (Figure 17b).

Due to technical issues, the KOR and OXTR were measured only in the prefrontal cortex and in the hypothalamus. Actually, the signals derived from KOR and OXTR antibodies in hippocampal tissue extracts did not produce an unequivocal interpretation. We performed Western blot analysis by using different methods of evaluation (primary and secondary antibody dilution, blocking solution (nonfat dry milk, or BSA), membrane (PVDF or nitrocellulose), in order to improve the quality of the analysis, without obtaining however clear or reliable results.

## 3. Discussion

As reported in the Introduction, an extensive literature states that during the perinatal period, intense emotions and associated variations in the hormonal milieu of the mother can have a fundamental role in shaping the neurophysiologic systems of the offspring. Premature and abnormally intense activation of the stress axis, during early ontogenetic windows in the offspring [7], have been shown to represent a predisposing factor towards vulnerability to the development of psychiatric symptoms such as those characterizing conduct disorder and autism spectrum disorder [8,9,10,11]. With the aim of developing an experimental preclinical model, we studied the male mouse offspring during adulthood for changes in the HPA axis function and the response to acute stress, comorbid for profiles of social competence, and emotional contagion [43,48]. We complemented the study with the assessment of the modulatory in vivo effects of intranasal administration of oxytocin [43]. Another aspect that has been taken into consideration was a molecular investigation of the gene expression for glucocorticoid, mineralocorticoid, μ-opioid, κ-opioid and oxytocin receptors in behaviourally relevant brain areas.

### 3.1. Titers of Plasma Corticosterone and Its Receptors in Brain Areas of Adult Offspring 

Although the use of hormonal supplementation only mimics a single aspect of stress, exogenous corticosterone was administered to mouse dams either during late gestation or early lactation. Data obtained from the present study confirmed that rodents that received high doses of corticosterone during perinatal period showed aberrant activation of the HPA axis in adulthood in response to an acute stress [52,53,54]. This confirms previous literature [55] and can be explained through an abnormal negative feedback. The literature on this aspect is not however unequivocal, and mixed results are also reported, possibly as a function of differences in the nature of the stress, the length of exposure and/or the dosage of corticosterone administered, including the use of different rodent species and strains [23,43,54,56].

In order to assess molecular correlates of the physiological and behavioural profiles scored, GR and MR density in behaviourally relevant brain areas was investigated [57,58]. An analysis of the hypothalamus revealed a general decrease in the GR levels in both PreCORT and PostCORT subjects. This result is in agreement with a compensatory response to the marked elevation in plasma corticosterone released in response to restraint stress [59]. This profile was accompanied by a relevant increase in mineralocorticoid receptors in the same area (quite clear in PreCORT group). These data confirmed previous findings [57], that reported an altered MR/GR ratio, in individuals exposed to high levels of maternal glucocorticoids. Moreover, the fact that only PreCORT subjects showed marked changes is in accordance with behavioural analyses that reported more relevant traits of social skills modifications in this group.

With respect to the hippocampus, a decrease of MR accompanies the hypersecretion of basal corticosterone in the present study, thus confirming previous reports [60]. This profile was particularly evident in PreCORT subjects [61,62]. The picture of altered expression of mineralocorticoid receptors is coherent with the functional impairments of this brain area associated with typical traits of behavioural disorders [63,64,65]. These results were obtained in rodents, but several lines of evidence indicate that the same might happen in both non-human primates and humans [63,64].

Somewhat less intense were the molecular changes obtained in the PostCORT group. This picture could refer to the phenomenon of the stress hyporesponsive period (SHRP), which sees rat and mouse pups being naturally kept less responsive to environmental stress, during the first ten postnatal days [17,18]. Actually, a regular neurobehavioural development requires a general picture of low functional activation on both mother and offspring during the perinatal life of the latter. This phenomenon may at least partially account for the fact that PostCORT subjects did not show similar marked consequences as the PreCORT ones, which were administered aberrant concentrations of corticosterone as well.

With respect to the modulatory effects of oxytocin, intranasal administration led to consistent reduction of plasma corticosterone released under acute stress condition, which is in line with previous findings [43]. Notably, a specific increased sensitivity to oxytocin appeared in adult subjects belonging to the PreCORT treatment group, thus highlighting the strict regulatory role of oxytocin on the HPA axis function. Indeed, this profile is suggesting a correlation between perinatal treatment and adjustments in the oxytocin system. Past works showed how subjects exposed to stressful events were characterised by a variant allele of oxytocin receptors [46].

### 3.2. Profiles of Sociability and Modulation by Oxytocin

In preclinical investigations, gestational stress or corticosterone administration are reported to lead to persistent deviations in social performance and motivation [66,67]. The natural tendency of mice to acquire new information towards novel social companions for outbreeding and new resources for food [68,69] appeared to be largely exaggerated in PreCORT and PostCORT subjects: this profile might be referable as a persistence-during adulthood-of adolescent-like behaviour [63]. This “infantilization of behaviour” is coherent with previous studies on guinea pigs that had their mothers exposed to stressful conditions during gestation and the perinatal period [19,70,71]. In the same line [70], adult rats raised with stress experiences early in life were characterised by a reckless endangerment which is typical of young animals [69,72]. Persistence in adulthood of the irresponsible and childish attitude could get maladaptive because of potential exposure of animals to hazardous situations or even predators [69,73]. As a consequence, PreCORT and PostCORT subjects might be more prone to potentially high risk-taking activities [74,75].

With respect to the modulatory action of oxytocin. It is worth noticing how the drug exerted different effects, depending on the basal rate of the considered behaviour in the different experimental groups. In order to explain these differential results, we could make separate considerations. One aspect of social interaction involves the strengthening of bonds between already familiar individuals. This seems the case for the regular profile exhibited by AFR subjects. Other considerations can be made for PreCORT and PostCORT subjects, which were characterised by somewhat an abnormal sociability profile, showing themselves as aberrantly open to novel social stimuli. Oxytocin had a relevant compensatory effect. [43]. Again, and this time at a behavioural level, increased responsivity to drug effects was specifically associated with the PreCORT group.

### 3.3. Profiles of Emotional Contagion and Involvement of Oxytocin Regulation

Individuals affected by major psychiatric symptoms, as reported in the Diagnostic Statistical Manual (DSM-V) [27] show profound alterations of social domain, including a reduction of affective and cognitive aspects of empathy behaviour. With the aim of modelling normal and abnormal profiles, we relied on preclinical studies reporting the presence of behaviours referable to empathy in rodents [49]. In this framework, the analysis of the social modulation of pain response to a formalin hind-paw injection, has been carried out by means of the emotional contagion assay [48]. As reported in the Introduction, the empathic response (emotional contagion) has been shown to be inhibited under activated HPA axis function in both humans and animal models [34]. We relied on this approach to investigate the consequences in adult mice of the precocious manipulation of the HPA axis [23,27,76].

#### 3.3.1. Observers’ Point of View

A general reduction of rate of paw-licking-behaviour used as a reliable index of emotional contagion-was suggested by Observer mice from the PreCORT and PostCORT groups, which were characterised by an exaggerated response to stress. Such a finding appears quite in agreement with the reported deficient emotional contagion capacity, upon increased HPA axis activation [34]. Intranasal oxytocin efficaciously downregulated the stress response and helped to revert the deficient emotional contagion observed, especially in PreCORT subjects, in accordance and in extension of previous work [34,43].

It is difficult to reconcile the unique increased responsivity to the physiological and behavioural effects released by exogenous oxytocin, evidenced in the PreCORT group with the picture that emerged from the molecular investigation in brain areas. Indeed, contrary to our expectation, the oxytocin receptor levels resulted to be markedly increased in the prefrontal cortex and in the hypothalamus of PostCORT mice compared with other perinatal treatment groups. Although, we have no information on the receptor affinity for its ligand in these mice. It can be hypothesised that the abnormal stimulation of the HPA axis in the PostCORT group (namely, it occurred during the SHRP time window), also interacted with the development of OXT system. Postnatal CORT might have caused an oxytocinergic hypotonus [13], with an ensuing upregulation of oxytocin receptors in target regions of hypothalamic oxytonergic neurons, i.e., the hippocampus and prefrontal cortex. Indeed, Lesse and colleagues [77] reported that chronic postnatal stress programmed increased oxytocin gene expression in the hippocampus of adult mice [78]. Further, it could be hypothesised that the increased levels of plasma corticosterone evidenced in adult PostCORT subjects may come as a consequence of the reduction in levels of circulating OXT (however, not measured in this study), as an adaptation to the increase in OXTR in brain areas. Indeed, some clinical studies reported oxytocin receptor epigenetic variations or modifications in the circulating levels of OXT in individuals affected by autism and conduct disorders [79]. In the same line, there are reports in symptomatic mouse models [43,48].

As a whole, a disturbed profile was found in both physiology and behaviour of both PreCORT and PostCORT mice. The profile was however generally positively modulated by oxytocin administration, with differential efficiency in the two perinatal treatment groups. Notably, scoring the different postures adopted by Observer mice during the exhibition of paw-licking behaviour allowed us to unveil a preference for the adoption of the in-back posture towards the Demonstrator companion. A tentative explanation can consider that mice are preys-in nature-and accordingly they tend to hide any frank manifestation of pain-elicited behaviour, which is usually associated to a vulnerable phenotype. Further, it could be hypothesised that Observer subjects adopted the in-back posture more frequently to finely down-tune direct eye-contact with the conspecific in pain. To add further complexity to the picture, a sort of internal conflict has possibly emerged: on one side, an empathy-like response in front of the conspecific in pain, was facilitated by oxytocin [43]. On the other side, released emotional contagion-driven by oxytocin administration-may have resulted in excessive sensitivity to pain in the other (Demonstrator companion). According to a self-protective down-tuning urgency, the in-back posture was adopted more frequently.

With respect to self-grooming behaviour, also measured during the emotional contagion assay, data acquired from the Observers indicated elevated general involvement in this auto-directed activity. No significant or reliable changes due to perinatal treatment were found. To date, there are conflicting opinions regarding the function of this complex behaviour, with an evolutionary conserved sequencing pattern [80,81] In addition to being performed for hygiene reasons (i.e., cleaning their fur, paws, tail), some authors believe that it can be associated with anxiety levels [82]; other authors instead attribute to it a pro-social communicative role between conspecifics [83,84]. Furthermore, the activation of the emotional state of the Observers-released by the presence of a cage mate in pain and directly facilitated by oxytocin-led to an increase in self-grooming behaviour. Indeed, this behaviour is enhanced in a social context by indirect stimulation of endogenous oxytocin release [85,86,87]. Actually, pre-test administration of oxytocin significantly increased the rate of self-grooming in all subjects, which is in accordance with the reported oxytocin augmented rate following intracerebral injections in rats [85,86]. It can be hypothesised that both oxytocin and the social context, respectively activated neural circuits that promote grooming behaviour. Indeed, even allogrooming behaviour is thought to increase after exposure to a conspecific in pain [88,89,90].

#### 3.3.2. Demonstrator’s Point of View

Analysis of behaviours scored allowed to reveal that the Demonstrators seemed to have a marked awareness about the individual characteristics of each social companion they were interacting with. Namely, whether it was from AFR, PreCORT or PostCORT perinatal treatment groups. Demonstrator mice instead were all from the AFR group. We found that Demonstrators spent most of the time licking their hind paw in proximity to those Observers belonging to the AFR group. This increased preference may perhaps reflect the share of perinatal condition with the companion. Further, the inhibition of paw-licking behaviour presented by Demonstrator when in proximity to Pre and PostCORT companions, might be accounted by generally disturbed physiological and behavioural profile offered by these subjects.

Furthermore, the performance of Demonstrators, which were not administered any drug, appeared quite affected by the kind of treatment delivered to their companions, before testing. Indeed, Demonstrator mice exhibited increased paw-licking behaviour when found in proximity of Observers that were administered oxytocin, and specifically if these were from the AFR group. It is not easy to explain this result, but it can be hypothesised that oxytocin administered to Observer companions, might have actively reduced the stress associated with the context and thus orchestrated the channelling of physiological arousal, thus directly modulating their body postures and associated social signals [89]. This could have had an indirect anxiolytic effect [43] also on the Demonstrator companion.

With respect to self-grooming activity exhibited by Demonstrator mice, an increased rate occurred when these subjects were in proximity to AFR controls. It can be also noticed again that Demonstrator mice were apparently able to distinguish-somehow-an abnormal profile in both PreCORT and PostCORT companions. Indeed, when in the presence of subjects from these two groups, levels of spontaneous self-grooming activity were greatly reduced, as it was the case for paw-licking activity. Again, as previously hypothesised in the case of the Observers, the whole picture may be interpreted as a general inhibition released by the presence of both PreCORT and PostCORT abnormal companions [91].

### 3.4. Profiles of Pain Sensitivity and Effects of Oxytocin as a Function of Test Setting

We were also interested in investigating individual profiles of basal pain sensitivity and response to oxytocin modulation that could emerge as a function of the characteristics of test setting. Interestingly, mice from the three perinatal treatment groups, which exhibited differences in the emotional contagion assay (social context), did not show reliable changes, when assessed individually for basal sensitivity to pain, in the classical formalin test. As a whole, regardless influence of perinatal treatment, but in line with the reported “social modulation” of pain [48,49], a tendency towards socially increased response to pain resulted globally associated to testing in a social context. Oxytocin administration exerted an additional positive modulation on this profile by interacting with the characteristics of test setting. Indeed, it slightly increased exhibition of paw-licking by most groups upon a social context, while drug effects appeared negligible when assessed in a non-social context. In literature, oxytocin is reported to have null or some analgesic effect, usually observed if this drug is administered at high dosages [92,93]. In contrast in the present study, the precocious manipulation of the HPA axis allowed to unmask a drug-induced sensitization of paw-licking response in the PreCORT group, which was specifically released upon the social context. Indeed, oxytocin confirmed its pro “empathy-like” action, by facilitating exhibition of emotional contagion in a generally disturbed experimental group.

### 3.5. Molecular Analyses in Brain Areas of Adult Offspring

Abnormal stimulation of the HPA axis during two perinatal windows of vulnerability represents an important environmental variable with epigenetically induced alterations in terms of physiological and emotional responses [23,24,53,94]. Related changes occurred at a molecular level, in terms of receptor density among brain areas involved in the regulation of the stress response. As expected, such changes occurred both for glucocorticoid and mineralocorticoid receptors, since corticosterone shows an affinity for both the receptor types mentioned (for discussion on oxytocin receptors, see Section 3.3).

### 3.6. Modulation of Profiles of Opioid Receptors in Adult Offspring

Endogenous opioids actively participate through the three major brain opioid receptors (μ, κ, and δ), also in interaction with glucocorticoid hormones in the modulation of the responses to stress [90,95,96] as well as of a series of cognitive and emotional responses [97]. Social and affiliative behaviours are proposed to tightly depend on the level of endogenous opioid peptides, based notably on striking similarities between social attachment and drug addiction [98]. Activation of μ-opioid receptors serves positive mediation of social affiliation and play in both rats and mice during adolescence [72,99]. While antagonist agents negatively modulate social-prone interactions [50,72].

Present findings confirm the implication of the μ-opioid system in the regulation of the HPA axis; generally, through pro-opioid peptides [100,101,102,103]. In line with past studies on consequences of chronic stress [103,104,105], μ-opioid receptors were found to present profound modifications in both PreCORT and PostCORT subjects, with reduced density in the hippocampus, and the opposite situation in the hypothalamus [23]. A tendency to increased levels of MOR in prefrontal cortex were evidenced here in PostCORT subjects as well. The latter finding may suggest that hyperresponsiveness of the μ-opioid system in both PreCORT and PostCORT subjects mimicked the prolonged exposure to low doses of opioid agonists, justifying the general abnormal profile of social behaviour observed in these two groups. In addition, given the role of the hippocampus in the regulation of emotions and behaviour [34], the significant decrease in the expression of MOR in this brain area might help to explain the altered behavioural profile characterising the PreCORT subjects, and it is quite coherent with studies reported by Milner and colleagues [105]. In agreement, decreased levels of MOR were reported in several brain areas, including the hippocampus of adult rats exposed to stress during gestation [106].

An analysis was also conducted on κ-opioid receptors, which are reported to exert an opposite modulation of social behaviour, compared with μ-opioid receptors [50]. Actually, characterisation of changes in κ-opioid receptors in brain areas might add important information to the present framework. In the absence of changes in the hypothalamus, relevant variations were found in other brain areas. Due to the appearance of such receptors since the prenatal phase (they rapidly increase starting from E 14.5), corticosterone treatment administered to dams during late gestation and produced an organisational upregulation on κ-receptors, altering their expression in prefrontal cortex. It is possible that the abnormal sociability profile and levels of emotional contagion evidenced in PreCORT subjects could be accounted at least partially by the increased density of these receptors found in PFC [107]. κ-opioid receptors are known to be involved in the regulation of emotional and motivational states [108]. Physiological KOR activation usually brings to responses of aversion, especially during the perinatal phase [109]. KOR pharmacological stimulation, could lead to anhedonic-like behaviours, as well as stress disorders or asocial behaviour [110]. In this framework, κ-opioid antagonists are actively studied with reference to major depression and anxiety disorders.

## 4. Materials and Methods

### 4.1. Animal Rearing and Conditions

Pregnant outbred CD-1 mice (*n* = 22) purchased from Charles River^®^, Calco, Italy arrived at 13 ± 1.5 days of pregnancy. Females were randomly assigned to the following groups: AFR (Animal Facility Rearing), *n* = 9; Prenatal CORT, *n* = 7; Postnatal CORT, *n* = 6. Dams were individually housed in standard polycarbonate cages (33.0 *×* 13.0 × 14.0 cm) with sawdust bedding and standard metal tops as lid. They were maintained on a reversed 12:12 h light:dark cycle (lights off at 6:00 a.m.) with temperature at 21.0 ± 1.0 °C and relative humidity of 60.0% ± 5.0%. Both temperature and humidity were monitored and kept constant. Animals were provided rodent pellets ad libitum (Altromin-R, Rieper SpA); they were also provided environmental enrichment in the form of shelter material (Nestlets^®^, Ancare).

AFR dams had free access to tap water; Prenatal CORT (PreCORT) dams were supplemented with a corticosterone (Sigma, St. Louis, MO, USA) solution in a dose of 80 μg/mL in drinking water from pregnancy day (PD) 13 until delivery (PD 20)-they received tap water from lactation day (LD) 0. Postnatal CORT (PostCORT) dams received the same corticosterone hemisuccinate solution from LD 0 to LD 7, and then returned to tap water. The dose of corticosterone was chosen on the base of previous studies by Macrì and colleagues [44]. All animals were inspected daily for delivery at 9:00 a.m. and day of birth was designated as postnatal day (PND) 0. Litters were not culled.

### 4.2. Experimental Timeline

Experimental subjects were split into two “arms” (see Figure 1).

Arm 1 was dedicated to the Behavioural battery, since all the subjects underwent a series of social tests during adulthood. Four male subjects from each litter were assigned to arm 1. At the end of all behavioural tests, adult subjects belonging to Arm 1-excluding Demonstrators-were assessed for HPA stress reactivity.

**Figure 18:**
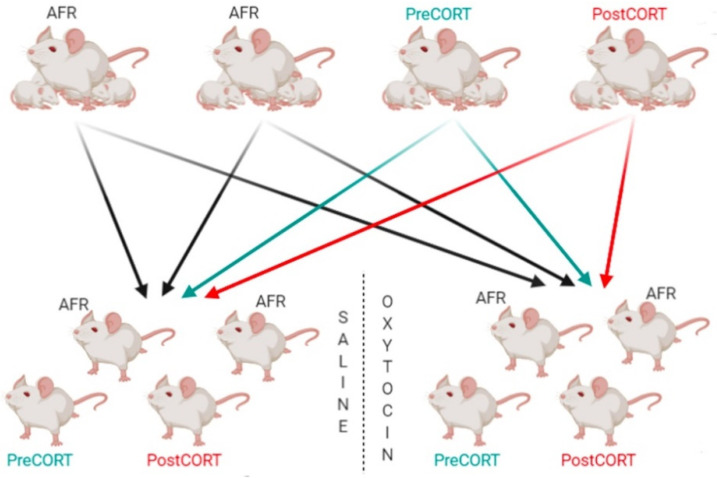
Rearrangements at weaning. Schematic illustration of family rearrangements at weaning, for arm 1. Male subjects were rearranged in order to obtain pairs of identical 4-mice groups, randomly assigned either to SAL Lane or OXT Lane. Each group consisted of 2 AFR, 1 PreCORT and 1 PostCORT subjects.

One of the aims of this experimental design was to study the modulatory effects of oxytocin, thus Arm 1 consisted in two parallel “lanes” of pharmacological treatment. Each lane consisted in 8 rearranged groups of four animals: 1 PreCORT, 1 PostCORT, 2 AFR subjects; each group belonging to SAL Lane had a reciprocal identical group assigned to OXT Lane (Figure 18). This meant having pairs of groups rearranged with the same criterion: AFR subjects picked from the same two litters of origin; PreCORT from the same litter of origin; PostCORT from the same litter of origin (Figure 18). At weaning (postnatal day 19–20), all mice from different litters, perinatal treatment AFR, Pre-CORT, and Post-CORT groups were rearranged to form novel groups of four males (tqo AFR—one designated to act as Demonstrator in the Emotional Contagion test, the other as an Observer—one Pre-CORT and one Post-CORT). For experimental reasons, animals remained housed in groups of four from weaning (PND 19–20) until PND 80–83.

All groups belonging to arm 1—from both OXT and SAL Lane—underwent the following social tests:

Sociability test (PND 71–PND 76)—a classical social test that allows to score the preference for a social stimulus, as well as for social novelty.

Emotional Contagion assay (PND 80–PND 83)-developed by Laviola and colleagues [48] to score the inter-individual differences in empathy-like behaviour.

As a control experiment, the baseline pain reactivity, all mice that previously acted as Observers during the Emotional Contagion assay were also assessed for pain sensitivity in the Formalin test after at least five days of recover from the previous injection.

Arm 2 was referred to as the Molecular Arm and included the remaining males from the 22 litters used in this study. Subjects belonging to each perinatal treatment group (AFR, *n* = 9; PreCORT, *n* = 8; PostCORT, *n* = 9) were chosen for brain collection and molecular analyses in order to evaluate the following parameters: glucocorticoid receptors (GR), mineralocorticoid receptors (MR), μ-opioid receptors (MOR), κ-opioid receptors (KOR) and oxytocin receptors (OXTR). It has been chosen not to expose any of these subjects to behavioural tests in order to have “clean” animals to perform post-mortem analysis on brain areas of interest. This procedure allows an evaluation of the differences among the chosen parameters that is independent from the effects of any prior experimental procedure, except perinatal treatments.

### 4.3. Oxytocin Administration

Oxytocin (C_43_H_66_N_12_O_12_S_2_; MW 1007.19; Sigma-Aldrich S.r.l., Milano, Italy) was dissolved in saline (0.9% NaCl) and administered intranasally in a volume of 2 μL to each mouse in dose of 20.0 μg/kg (12.0 IU/kg). Other mice received the same volume of saline (vehicle). It has been assessed to use a low volume in order to facilitate active inhalation. The dose and the volume chosen are based on previous studies that reported efficacy on the modulation of social behaviour, without leading to any effect on the peripheral nervous system [43,111,112,113]. Solution was administered in two drops of 0.5 μL for each nostril using a 2 μL Gilson pipette. The intranasal administration procedure required mice to be mildly restrained, to allow for movements related to active inhalation. All mice were habituated to this procedure during the three days preceding the first test (Sociability test). Each drop, after being gently placed on the nostril, was reflexively inhaled. After administration, mice were returned to their home cage and acute effects were assessed approximately 10 min following the administration [43].

### 4.4. Sociability Test

Between PND 71-PND 76 mice belonging to Arm 1 were tested for sociability using the Sociability test [114]. The whole paradigm consisted of three episodes, each lasting 10 min.

- Episode 1—Habituation Phase: Mice were individually placed in a black Plexiglas^®^ box (20 *×* 40 cm^2^ and 40 cm height) containing two identical empty wire cups (10 × 10 cm^2^ and 11 cm height; pencil holder, IKEA) placed close to the two opposite corners of the apparatus. During this phase, no social stimuli were present, and the experimental mice were free to explore the entire arena.
- Episode 2—Social Preference: One unfamiliar male mouse was placed under a wire cup, serving as social stimulus (Stranger 1), whilst the other cup remained empty. The position of the social stimulus was counterbalanced across subjects. The preference for the cup containing the social stimulus (Stranger 1) over the empty cup was evaluated. In addition, further analysis concerned the interaction with the social stimulus only.
- Episode 3—Social Novelty Preference: An additional unfamiliar male mouse (Stranger 2) was placed in the previously empty cup, whilst the other cup contained the mouse encountered during Ep. 2 (Stranger 1). The preference for the cup containing the novel social stimulus (Stranger 2) over the cup containing the familiar social stimulus (Stranger 1) was evaluated. Moreover, further analysis concerned the interaction with the novel stimulus only.

Mice that acted as strangers were unfamiliar same-age CD-1 male mice not belonging to any of the two Arms in the experimental design; previously habituated to placement in the cup. The habituation phase lasted 10 min for three consecutive days. Test mice were administered either oxytocin or saline 10 min before the beginning of the test which was performed minimizing gradients in light, temperature, sound and other environmental conditions. In order to avoid positional effects, the position of the cups and the Stranger mice were counterbalanced across sessions. The apparatus was cleaned with 30% ethanol/water solution at the end of each test session. The experiment was video-recorded (Sony DCR-SX21E) and the behavioural patterns exhibited by AFR, PreCORT and PostCORT mice were scored using an ethological software (The Observer XT 15-Noldus, Wageningen, The Netherlands) by a single operator. The ethogram comprised the following behaviours: time spent in close proximity to the cups and self-grooming during the three phases; and locomotion during the habituation phase.

### 4.5. Emotional Contagion Assay

To investigate inter-individual differences in empathy-like behaviour, between PND 80-PND 83 mice belonging to arm 1 underwent the Emotional Contagion test previously described [43,48]. In this test, four familiar mice were tested at the same time, with a total of 16 subjects acting as Demonstrators and 48 subjects acting as Observers.

**Figure 19:**
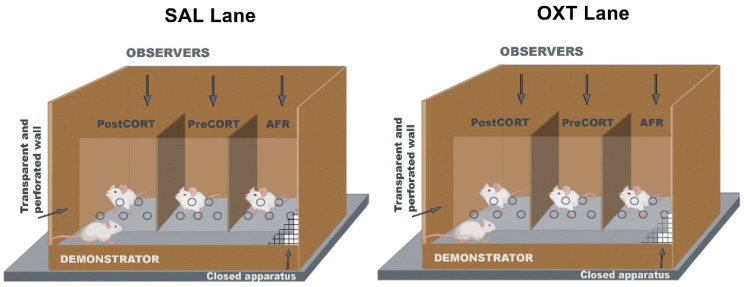
Emotional Contagion assay setting. Observers’ side and Demonstrator’s side were divided by a transparent and perforated Plexiglas^®^ partition, while the Observers’ side itself was divided into three equal sectors by opaque walls [48].

This paradigm allowed to evaluate the extent to which a painful experience can be socially transmitted between individuals [43,48]. Subcutaneous injection of a small volume of formalin-diluted vehicle into the plantar surface of the animal’s hind-paw was used as a painful stimulus [115,116]. This procedure caused a behavioural pain response in the form of paw-licking behaviour [117,118]. The test was performed in a custom-made apparatus consisting of an opaque plastic box (20 *×* 20 cm^2^ and 30 cm height) divided in two equally sized sectors (Demonstrator’s side and Observers’ side) by a transparent and perforated Plexiglas^®^ partition allowing olfactory, acoustic and visual communication between the Demonstrator and the Observers (Figure 19). The Observers could not see each other because of the presence of opaque plastic walls dividing the Observers’ side in three compartments. The animals were habituated to the apparatus by being placed in their respective compartment for 10 min per day during three consecutive days before testing. On the fourth day, according to the rationale and procedure described by Laviola and co-authors [48], mice were injected with 25 μL of a formalin solution in a hind-paw and then gently placed into the assigned compartment for 40 min. Notably, it has been used a standard formalin concentration (1%) for the Demonstrator mouse and a very low formalin concentration (0.2%) as a primer for the Observer mice. In order to assess the effects of drug administration, Observer mice received either oxytocin or saline 10 min before the beginning of the test-according to the Lane in the experimental design they belonged to. The test was performed minimizing gradients in light, temperature, sound and other environmental conditions. Moreover, in order to avoid positional effects of interaction between the Demonstrator and the Observers, the position of the latter (i.e., left vs. centre vs. right) was randomized throughout sessions, with respect to the perinatal treatment group they belonged to. Between sessions, the apparatus was cleaned with 30% ethanol/water solution. The test was video recorded (Sony DCR-SX21E) and the duration of paw-licking behaviour, touching (nose pokes through the hole on the clear separator), and self-grooming-in both Demonstrator and Observer subjects-were scored by a single operator using a dedicated software (The Observer XT 15-Noldus, Wageningen, The Netherlands).

As a refinement of behavioural analysis already performed in previous studies [43,48]. paw-licking behaviour was scored taking into account the body posture adopted by the Observer mice, namely in-front of the Demonstrator (in-front posture) or in-back to the Demonstrator (in-back posture). Additionally, as the Demonstrator mouse could adopt different local positions within the large compartment of the apparatus to it assigned: (i) in front of the AFR Observer, (ii) in front of the PreCORT Observer and (iii) in front of the PostCORT Observer. This parameter has been taken into account for all the behaviours exhibited by the Demonstrator mouse.

### 4.6. Pain Sensitivity in the Formalin Test

All mice that acted as Observers during the Emotional Contagion test, were subjected to a classical chemical pain test—between PND 86-PND 90—in order to assess their pain response. The Formalin test evaluates baseline chemical pain reactivity following the subcutaneous injection of formalin in the hind-paw in a non-social condition [115,117].

Previous studies used this test as a control procedure for the possibility that inter-individual differences in the social transmission of pain were explained by differential pain sensitivity [48]. The present study focused on the differences in pain sensitivity linked to the context (social vs. non-social) the experimental subjects were exposed to. In this regard, subjects were injected with 25 μL of the same formalin dosage (0.2%) they received during the Emotional Contagion test. The injection was performed in the plantar region of the contralateral hind-paw to that injected to the aims of the Emotional Contagion assay. Since all the subjects experienced at least five days of recovery from the previous injection, the latter was not expected to affect the behavioural results reported in the Formalin test. In addition, 10 min prior to the test, mice received intranasal administration of oxytocin or saline. Behaviours were scored using a dedicated software (The Observer XT 15-Noldus, Wageningen, The Netherlands) by the same operator who had previously scored the Emotional Contagion assay.

### 4.7. Restraint Stress and Assessment of Plasma Corticosterone

In order to investigate underlying differences in HPA-axis function and the modulatory effects of oxytocin administration thereon, all mice that acted as Observers during the Emotional Contagion assay (Arm 1) were investigated for physiological response to an acute restraint stress between PND 91–PND 98.

Mice were carried, within their home-cage, by a familiar experimenter to an adjacent room; blood samples were collected (t0) by retro orbital bleeding. The time elapsed between the experimenter entering the room and the completion of baseline blood sampling was less than 3 min. Mice were administered either oxytocin or saline and placed in a transparent Plexiglas^®^ restraint tube (2.8 cm diameter) for 25 min to induce a stress response. At the end of the stress exposure, a second sample was collected (t25), and then mice have been relocated to their home-cage. Additional samples were taken 35 min (t60) and 95 min (t120) later. For each blood sampling approximately 20 μL were collected into prechilled EDTA-coated tubes (Microvette^®^ Sarstedt, Nümbrecht, Germany). The procedure was performed between 8:00 and 11:00. Samples were cool centrifuged (2500 rpm for 20 min) and the plasma stored at −80 °C until assayed. Corticosterone was measured using a commercially available Corticosterone Enzyme-Linked Immunosorbent Assay (ELISA) kit (My BioSource, Inc., Atlanta, GA, USA).

### 4.8. Molecular Analyses from Adult Offspring’s Brain Areas

Subjects belonging to arm 2 were sacrificed between PND 87 and PND 90. Brain samples were collected in order to characterise the expression of glucocorticoid receptors (GR), mineralocorticoid receptors (MR), µ-opioid receptors (MOR), κ-opioid receptors (KOR) and oxytocin receptors (OXTR). Analyses were performed on the following brain areas of interest: prefrontal cortex, hypothalamus, hippocampus. Due to technical issues, KOR and OXTR were measured only in the prefrontal cortex and in the hypothalamus.

#### Western Blot Analysis

Briefly, samples of mouse brain (Prefrontal Cortex, Hypothalamus, Hippocampus) were placed in cold buffer (4 °C) RIPA buffer (50 mM Tris; 150 mM sodium chloride; 1 mM EDTA; 1% sodium deoxycholate; 1% Triton X-100; 0.1% sodium dodecyl sulphate (SDS); pH 7.4) supplemented with a protease inhibitor cocktail (Thermo Fisher, Monza, Italy). Brain tissues were sonicated and centrifuged at 10,000× *g* for 20 min at 4 °C. The supernatants were stored at −80 °C till the day of the experiment. The total protein concentration was determined using a Pierce™ BCA Protein Assay Kit (Thermo Fisher, Monza, Italy). Twenty micrograms (20 μg) of brain tissue were separated using Bolt 4–12%, Bis-Tris Mini Protein Gel. The separated protein bands were then transferred onto 0.45 μm PVDF membranes, saturated with 5% fat-dry milk for 1 h at room temperature (RT) and, finally, incubated overnight at 4 °C with primary antibodies against: glucocorticoid receptor (1:1000-Genetex, Italy), mineralocorticoid receptor (1:1000-Genetex, Italy), μ-opioid receptor (1:1000, GeneTex, Italy) and β-actin (1:5000-Thermo Fisher, Italy). Incubation with secondary antibodies peroxidase-conjugated was performed for 1 h at RT (Goat anti-Mouse IgG or Goat anti-Rabbit 1:5000-Thermo Fisher, Monza, Italy). Membranes were developed using the enhanced chemiluminescence (ECL Amersham-Little Chalfont, UK) by the chemiluminescence imaging system iBright CL1500 (Life Technologies, Bleiswijk, The Netherlands). Densitometric analysis of the bands was performed by ImageJ software. Each reported value was derived from the ratio between arbitrary units obtained by the protein band and the respective β-actin (chosen as housekeeping protein).

### 4.9. Statistical Analysis

Acquired data were analysed using the analysis of variance (ANOVA) with Perinatal treatment (three levels: AFR, PreCORT, PostCORT) and drug (two levels: oxytocin, saline) as between-subjects factors and repeated measures as within-subjects factor. In the presence of a significant ANOVA result on the main effects, post hoc analysis was not necessary. When the *p*-value of the interaction resulting from the ANOVA was ≤0.1 (Wilcox, 1987), multiple post hoc comparisons were made using Tukey HSD test. All statistical analyses were performed using SPSS^®^ Statistics software (IBM^®^, USA). All data are expressed as mean ± standard error of measurement (SEM). Outlier values, considered as values outside the reference range (average ± 1.96 *×* SD), have always been excluded. In the figures the symbol * has been used to indicate a significant main effect (* *p* ≤ 0.05) in the ANOVA analysis or a significant comparison in the Tukey test (* *p* ≤ 0.05); error bars indicate ± 1 SEM. For Western blot analysis, data are presented as mean ± SEM. One-way ANOVA was used, and Tukey’s post hoc multiple-comparison procedure was used. *p* < 0.05 was considered significant.

## 5. Conclusions

The results over the last decade have provided evidence suggesting that HPA axis dysfunction is not a simple consequence or an epiphenomenon of mental disorder, but on the contrary, it is a risk factor predisposing to the development of psychopathological behaviour. This susceptibility can be programmed through early-life events-starting even as early as in prenatal development. The high degree of neuroplasticity during early developmental stages acts as a critical window of sensitivity, allowing childhood adversity to convey vulnerability to mental illness in later life [3,116].

We confirmed and extended by means of a translational model, experimental evidence of comorbid changes in social and emotional domain in individual mice presenting exaggerated response to stress. This finding has been accompanied by functional analysis that evidenced how physiological alterations concerning the stress response have direct consequences on the way individuals interact socially with their conspecifics. Indeed, increased sensitivity to social stress has as a direct consequence a deficient social-prone behaviour, as previous work had pointed out in both humans and rodent model [34]. Intranasal oxytocin administration, which is known for its anxiolytic effects [119], managed to inhibit the aberrant physiological activation and behavioural response. Thus, confirming previous investigations in experimental models [43], as well as its role as a prosocial neuropeptide (see, e.g., response of AFR controls). For a summary of these effects, see Table 1 and Table 2.

Fine-grained behavioural analysis considering all subjects involved in the emotional contagion assay, both the Demonstrator and the Observer companions, evidenced how “empathy-like” behaviour is influenced in both directions. The administration of oxytocin allowed to observe direct modulatory effects on administered Observer mice; but also, an indirect influence on Demonstrator companions. The latter phenomenon took probably place through changes in subtle physiological and behavioural cues emitted from drug-administered subjects, and possibly perceived and recognized by the social partner.

Companion marked alterations at receptor level for GR and MR, as well as oxytocin and opioid systems were also evidenced in brain areas of behaviourally symptomatic subjects. Notably, the former occurred as a persistent organizational consequence of the increased stimulation of HPA axis function early in development. Interestingly, the direction of these changes was function of the specific brain area and of the selected phase of perinatal development targeted by abnormal HPA axis stimulation. We added here a direct comparison of the effects of prenatal and postnatal exposure to maternal glucocorticoids [23,24,54]. For a summary of these effects, see Table 3.

The picture of changes reported for OXTR density in brain areas is difficult to reconcile with the unique elevated responsivity to effects of exogenous oxytocin found in PreCORT mice. On the other hand, the PostCORT group only slightly benefited from the drug administration, mainly in terms of some reduction of the physiological stress response and, indeed, generally resulting in treatment resistance. Therefore, mapping the expression of OXTR in the animal/human brain, as well as clarifying the effects of OXTR genotype and epigenetic signatures, are critical to identify factors that predict personalised responsiveness to oxytocin at the individual level. Again, these translational results may be informative for clinical studies that include the use of oxytocin as a treatment for psychiatric conditions highlighting the need to identify subjects that could benefit of this drug in an effective way [120,121,122].

## Figures and Tables

**Table 1:** Summary of the effects due to oxytocin administration on physiology and behaviour. AFR, PreCORT and PostCORT (*n* = 8). Symbol * indicates a statistically significant increase (↑) or decrease (↓).

Parameter	Perinatal Treatment		
	AFR	PreCORT	PostCORT
Response to stress	=	↓ *	↓
Sociability (with Social Stimulus)	↑	↑	↑
Sociability (with Novel Stimulus)	↓	↓	↓
Emotional Contagion (Paw-Licking In-Front)	=	↑	=
Emotional Contagion (Paw-Licking In-Back)	=	↑ *	↑
Self-Grooming	↑	↑	=
Hole Probe Behaviour	↓	↓	=
Formalin Test (Paw-Licking in Non-Social Context)	↑	↑	↑

**Table 2:** Summary of the effects due to perinatal treatment on receptor density among three different areas of interest: prefrontal cortex, hypothalamus and hippocampus. Symbol * indicates a statistically significant increase (↑) or decrease (↓) in comparison with AFR subjects.

Receptor	Brain Area	Perinatal Treatment	
		PreCORT	PostCORT
GR	Prefrontal Cortex	=	↓
	Hypothalamus	↓ *	↓ *
	Hippocampus	↑ *	=
MR	Prefrontal Cortex	↑	↑
	Hypothalamus	↑ *	↑
	Hippocampus	↓ *	↓
MOR	Prefrontal Cortex	=	↑
	Hypothalamus	↑ *	↑ *
	Hippocampus	↓ *	↓
KOR	Prefrontal Cortex	↑ *	=
	Hypothalamus	↓	=
OXTR	Prefrontal Cortex	↑	↑ *
	Hypothalamus	=	↑ *

**Table 3:** Summary of the effects due to perinatal treatment on physiology and behaviour. Comparison between saline-treated PreCORT and PostCORT subjects (*n* = 8 for each group) and saline-treated AFR subjects (*n* = 8).

Parameter	Perinatal Treatment	
	PreCORT	PostCORT
Response to stress	↑	↑
Sociability (with Social Stimulus)	↓	=
Sociability (with Novel Stimulus)	↑	↑
Emotional Contagion (Paw-Licking In-Front)	↑	↑
Emotional Contagion (Paw-Licking In-Back)	↑	↑
Self-Grooming	↓	↑
Hole Probe Behaviour	↑	↓
Formalin Test (Paw-Licking in Non-Social Context)	↓	↓

## Data Availability

Original data will be shared upon reasonable request to the corresponding author.
